# Subclassification of second-degree tears at delivery: creation and reported outcomes

**DOI:** 10.1186/s12884-025-07371-z

**Published:** 2025-03-11

**Authors:** Eva Uustal, Malin Edqvist

**Affiliations:** 1 Department of Clinical and Experimental Medicine, Department of Obstetrics and Gynecology, Linköping University, Linköping, Sweden; 2 Department of Women’s Health, Karolinska Institutet, Department of Women’s Health and Allied Health Professions, Karolinska University Hospital, Stockholm, Sweden

**Keywords:** International classification of diseases, ICD-10-SWE, Obstetric labor complications, perineum, pelvic floor

## Abstract

**Background:**

Perineal tears at delivery are common. The current WHO classification system compacts all the varying extents of second-degree tears into one code. Some tears lead to long-term injuries. The correct identification and classification of disease is necessary for correct clinical management as well as for research. Regulatory standards govern care practices. This article describes the process of creating and testing new subclassifications for second-degree tears at delivery.

**Methods:**

The development and implementation of new subclassifications of second-degree perineal tears after delivery in Sweden are described. The new classification was tested for incidence and relevance via the national perineal laceration register (PLR) in 11,203 women with prospectively recorded second degree tears.

**Results:**

Second-degree tears after delivery are subdivided into four subgroups according to the anovaginal distance and the extent in length and depth of the largest perineal/vaginal tear, which can be combined with uni-or bilateral levator ani avulsion. Women with larger second-degree tears were more likely than women with smaller tears to report complications after eight weeks (OR 1.41 CI 1.21–1.64, *p* < 0.001) and one year (OR 1.27, CI 1.1–1.46, *p* < 0.001).

**Conclusion:**

Detailed subclassifications of perineal and vaginal tears are implemented in the Swedish ICD-10 coding system and Swedish national registers. The outcomes after second-degree tears differ according to their extent, which corroborates the classification rationale. These subclassifications can be used in studies of preventive measures, treatment and patient-reported outcomes and experiences taking into account the extent of second-degree perineal tears at delivery.

**Trial registration:**

Data regarding women were prospectively collected from the National perineal laceration register (PLR) from January 1, 2021, to December 31, 2022.

**Supplementary Information:**

The online version contains supplementary material available at 10.1186/s12884-025-07371-z.

## Highlights

Subclassification of second- degree tears at delivery have been established.

Women with larger tears have larger suturing times and worse patient-reported outcomes than women with smaller tears do.

**Supplementary Information:**

The online version contains supplementary material available at 10.1186/s12884-025-07371-z.

## Background

Second-degree tears are common and can cause substantial morbidity [1]. More women experience perineal pain after a second-degree tear or an episiotomy than after an intact perineum or first‐degree tear [2]. The extent, and possible consequences, of second-degree tears vary from small tears in the fibrous perineum to large muscle avulsions. Perineal and vaginal tears that involve muscle attachments contribute to sexual dysfunction and are associated with an increased risk of symptomatic pelvic organ prolapse later in life, particularly rectocele [3]. Injuries affecting the anal sphincter are sometimes wrongly classified as second‐degree tears and are therefore not diagnosed and sutured correctly [4, 5]. A perineal tear that is not found will not be repaired. The diagnostic process is influenced by systems, cognitive abilities, teamwork, and social factors that may either enhance or reduce diagnostic accuracy [6]. If an ailment or injury is sought after by official agencies, the importance of a thorough diagnostic procedure can be highlighted.

This article describes the process of creating subclassifications of second-degree tears at delivery. The second-degree subclassification was subsequently tested for differences in outcome via the perineal laceration register (PLR).

The text has been written according to the SQUIRE guidelines for quality improvement studies [7].

## Methods

This is a descriptive account of how a subclassification of second-degree perineal delivery tears was created and tested. A comprehensive national audit about prevention, diagnostic procedures, suturing, and follow-up routines after perineal tears was performed from 2013 to 2014 in Sweden. This multiprofessional national audit revealed a need for training among doctors and midwives regarding both the anatomy and classification of perineal tears, corroborating international data [8]. A multidisciplinary collaboration, the Pelvic Floor Education Group (PEG), was established by the SFOG (Swedish Society of Obstetricians and Gynecologists) and the Swedish Association of Midwives. Two obstetricians, two urogynecologists and three midwives, were appointed as experts and authors. The PEG collected excellent practice and care routines from the national audit, and a systematic literature review was performed [9]. A pelvic floor educational web program with tutorials about prevention, diagnostic procedures and treatment of perineal tears was created and finally published on July 1, 2017. The program is updated yearly. The process is further described on the program website [10].

As we created the educational framework for perineal tears, the members of the PEG identified the disturbing lack of precision in the classification of second-degree tears. There is a difference between small second-degree tears involving only the fibrous tissue in the perineum and large second-degree tears involving deep paravaginal muscles and fasciae, in terms of treatment and outcome for women. This was not reflected in the earlier classification system.

The PEG developed a new subclassification based on clinical experience over several meetings. We did not want an overly ambitious complex classification system detailing every anatomic structure in uni- and/or bilateral tears. Our pragmatic approach was that the longer and deeper the tear is, the more pelvic floor structures are likely to be involved. This concept was used in a previous study [11]. A three subtype-model according to the length and depth of the longest tear was discussed by the group as part of a first draft in 2018. The model was used in a clinical study, which revealed that measuring tears in centimeters was feasible for teaching and repeating [2]. We also wanted to implement mandatory bidigital anorectovaginal examination of all delivered women, and the classification was expanded to a four-subtype model to include the palpated anovaginal distance to quantify the thinnest part of the perineal body [12, 13].

The four-subtype model was published as a preliminary classification on the program website in 2018. It was introduced by the PEG group in national lectures and meetings and revised according to suggestions from stakeholders, obstetricians and midwives. It was found important to also allow future classification of levator ani avulsions or deep tears, unilateral or bilateral, and these classifications were added to the subclassification in late 2018 (Table 1).

**Table 1:** Second-degree perineal and vaginal tears at delivery, Swedish subclassification established 2020

Label	ICD Code	Description in the 2020 Swedish classification, clarification in italics	Proportions grade II perineal tears in the PLR	Classification code/label according to the WHO-classification ICD-11
**Second degree tear**	**O70.1**	Tear of perineal muscles/ muscle insertions but not anal sphincters. *Includes extension of a perineotomy. Excludes isolated vaginal tear (O71.4) The shape of the vagina is affected.*	N = Total 11,203	JB09.1 s degree perineal laceration involve, in addition, the fascia and muscles of the perineal body but not the anal sphincter
Small second-degree tear	O70.1a	Perineal tear affecting less than half the perineal body. *The anovaginal distance¤ is more than one cm¤. The tear affects the bulbocavernosus*, *transverse perineal muscles or their insertion points and can include a vaginal tear less than two cm deep*.	50% (5620)	No
Intermediate second-degree tear	O70.1b	Perineal tear affecting more than half the perineal body. *The anovaginal distance¤ is less than one cm. The tear affects the bulbocavernosus*, *transverse perineal muscles or their insertion points. Can include a vaginal tear less than two cm deep.*	1% (112)	No
Large second degree-tear with a low vaginal tear	O70.1c	Perineal tear affecting the perineal body and a vaginal tear up to or four cm long, more than two cm deep. *The tear affects the bulbocavernosus*, *transverse perineal muscles or their insertion points and the rectovaginal fascia.*	26% (2899)	No
Large second degree-tear with a high deep vaginal tear	O70.1d	Perineal tear affecting the perineal body and a vaginal tear more than 4 cm long, more than 2 cm deep. *The tear affects the bulbocavernosus*, *transverse perineal muscles or their insertion points and the rectovaginal fascia.*	1% (135)	No
Unspecified second-degree tear	O70.1X	Other unspecified second-degree tear	22% (2437)	JB09.Z Perineal laceration during delivery, unspecified
Unilateral levator ani tear	O70.1e	Unilateral avulsion or tear of the levator ani muscles. *Unilateral tear of the levator ani muscle complex ventral to the perineal plane. The muscle is avulsed from the symphysis pubis or fragmented*.	No data	No
Bilateral levator ani tear	O70.1f	Bilateral avulsion or tear of the levator ani muscles. *Bilateral tear of the levator ani muscle complex ventral to the perineal plane. The muscle is avulsed from the symphysis pubis or fragmented*.	No data	No

Distribution regarding extent of tears from the Perineal laceration register (PLR) in 2021 and 2022, and current WHO ICD-codes

Tear-depth is measured at a right angle from the vaginal wall

Tear-length is measured in the mucosal plane

Only the largest tear is classified but levator ani injury codes can be added separately

Explanatory text in italics

^¤^The anovaginal distance is the bidigitally palpated shortest distance between the anal canal and the distal vaginal wall

In late 2018, the classification was presented to the National Board of Health and Welfare, which in turn appointed a separate reference group of midwives, obstetricians, and generic classification experts to analyze and ratify the new subclassifications.

The perineal laceration register (PLR) is used by all Swedish delivery units for follow-up after obstetric anal sphincter rupture [14] and many units also use the PLR for follow-up after perineotomies and second-degree tears. Eight weeks after delivery, the women are asked for their subjective experience including complications such as pain, need for unplanned hospital visits and anal incontinence. After one year, the women are asked to also report their overall assessment with the result after the tear as well as several aspects of pelvic floor function. The PLR was described in detail earlier [15, 16] and the questionnaires are presented in the supplementary material. Registered medical record data for 2021 and 2022 were converted into new subclasses in PLR via an algorithm for subclassification. The new subclassifications after second-degree tears were then compared to each other according to the duration of repair, patient-reported complications at eight weeks and one year, and overall patient assessment of the results. All 11,203 women from hospitals using the PLR for systematic follow-up of all women after second-degree tears in 2021 and 2021 were included.

## Statistics

Descriptive data are presented as the means and standard deviations for continuous variables and as quantities and proportions for categorical variables. For comparisons between 2 groups, the Welch two-sample t test was used for continuous variables; for categorical variables, Fisher’s test was used to assess proportions. Risk estimates are presented as adjusted odds ratios (ORs) with 95% confidence intervals (CIs). All the statistical tests were 2-sided and had a p value < 0.05. The statistical analyses were conducted via the R statistical package (R version 4.3.0, 2023; R Foundation for Statistical Computing; Vienna, Austria).

## Results

On January 1, 2020, the new subclassification sets were published by the National Board of Health and Welfare classification department. The subclassification sets with added explanatory texts were published on the Pelvic floor education website and in the national diagnosis code manual published by the SFOG in 2020 [10, 17]. The subclassification is now implemented nationally.

Among the systematically registered second-degree tears, 78% could be subclassified via data from the current medical record system (Table 1). The questionnaire response rate for the PLR at the one-year follow-up was 69%, and it did not differ according to the extent of the tears.

**Fig. 1:**
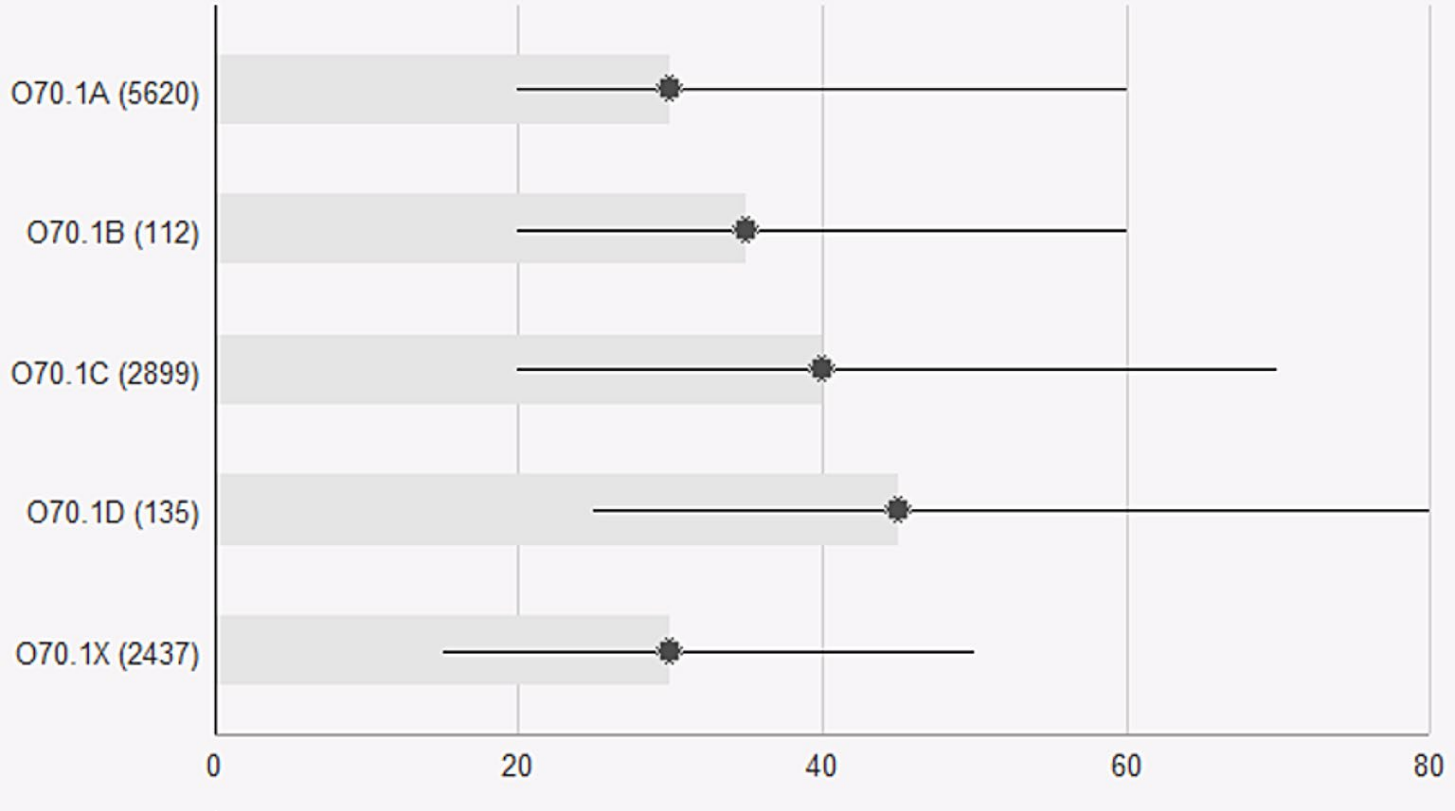
Operating time (minutes) for second-degree tears according to subclassification: O70.1a Small second-degree tear.O70. 1b Intermediate second-degree tear.O70.1c Large second degree-tear with a low vaginal tear. O70.1d Large second degree-tear with a high deep vaginal tear. O70.1x Unspecified second-degree tear. Perineal laceration register data from 2021 and 2022. Medians and 10th and 90th percentiles

**Fig. 2:**
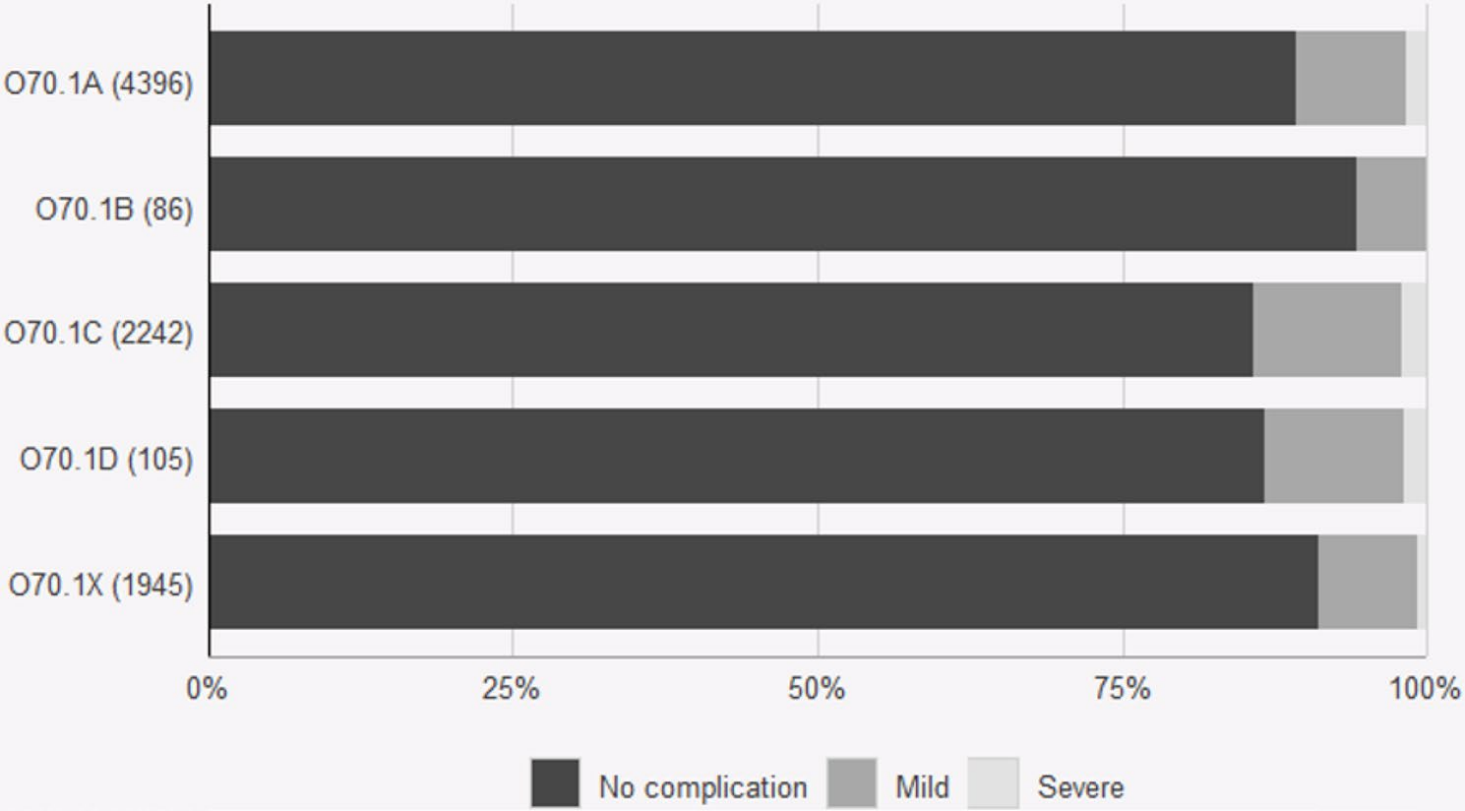
Patient-reported complications eight weeks after second-degree tears according to subclassification: O70.1a Small second-degree tear. O70.1b Intermediate second-degree tear. O70.1c Large second degree-tear with a low vaginal tear. O70.1d Large second degree-tear with a high deep vaginal tear. O70.1x Unspecified second-degree tear. Perineal laceration register data from 2021 and 2022. Medians and 10th and 90th percentiles

**Fig. 3:**
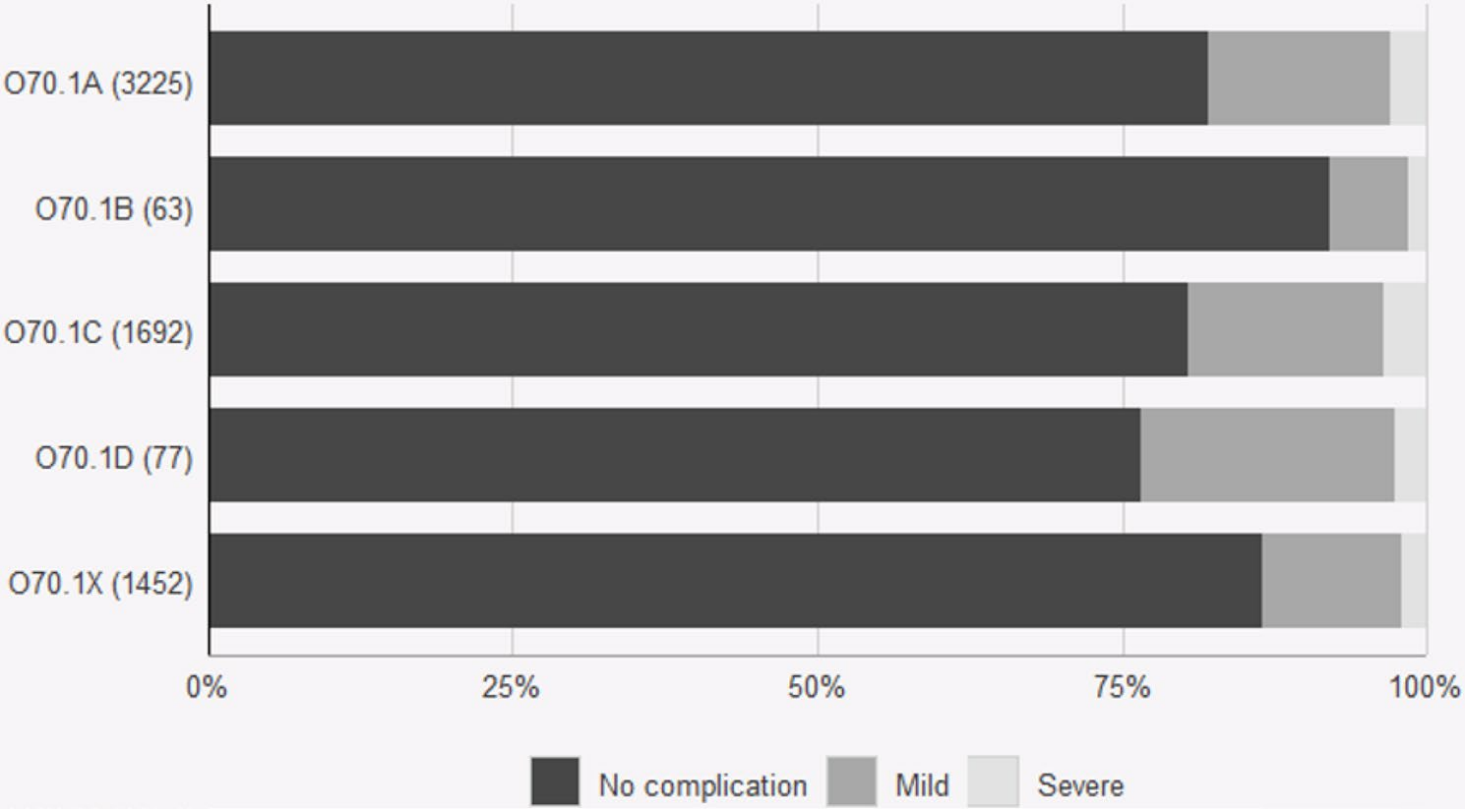
Patient-reported complications one year after second-degree tears according to subclassification: O70.1a Small second-degree tear. O70.1b Intermediate second-degree tear. O70.1c Large second degree-tear with a low vaginal tear. O70.1d Large second degree-tear with a high deep vaginal tear. O70.1x Unspecified second-degree tear. Perineal laceration register data from 2021 and 2022. Medians and 10th and 90th percentiles

**Fig. 4:**
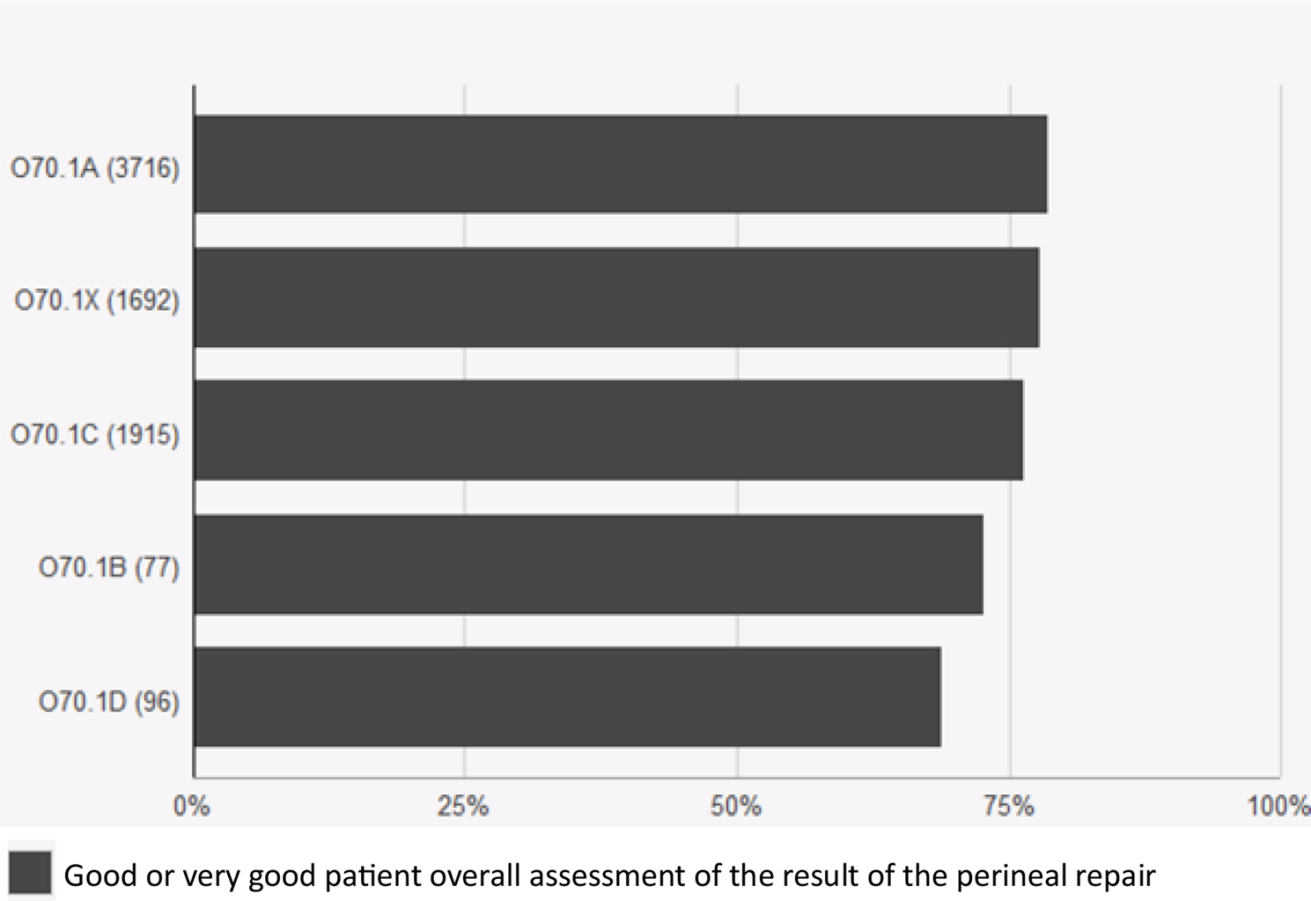
Patient overall assessment of the results of second-degree perineal repair according to subclassification: O70.1a Small second-degree tear. O70.1b Intermediate second-degree tear. O70.1c Large second degree-tear with a low vaginal tear. O70.1d Large second degree-tear with a high deep vaginal tear. O70.1x Unspecified second-degree tear. The black boxes denote the proportion of women reporting good or very good assessments one year postpartum. Perineal laceration register data from 2021 and 2022

The subclassified tears were tested for relevance via data from the PLR. The 22% of women with tears that could not be classified due to lack of data regarding bidigital palpation (O70.1X) had durations of suturing and complication rates similar to those of women with smaller tears (O70.1 A and B) (Figs. 1, 2 and 3). The second-degree tears were dichotomized into larger (O70.1 C and D) and smaller tears (O70. X, A and B) to allow calculation of the odds ratio. Women with larger tears were more likely than women with smaller tears to report complications after eight weeks (OR 1.49 CI 1.29–1.72, *p* < 0.001) and one year (OR 1.27, CI 1.1–1.46, *p* < 0.001). (Figures 2 and 3). Overall satisfaction with the outcome one year after perineal tear was lower among women with larger tears (OR 0.87 CI 0.76–0.99 *p* = 0.029) (Fig. 4). The duration of suturing was longer for women with larger tears than for women with smaller tears (Fig. 1). The current Swedish medical record system does not yet systematically record levator ani avulsion injuries at delivery so these codes were not available for analysis in the time period.

## Discussion

The understanding of which specific structures are involved in obstetric tears has expanded over the last few decades. The classical concept of externally visible superficial perineal tears has evolved, and new diagnostic measures and codes now also target injuries of deeper pelvic floor structures. Our study shows that the implementation of mandatory bidigital anorectovaginal examination in Sweden has been successful. The examination as instructed in the pelvic floor education web program was documented in 78% of all delivered women. This examination is crucial for establishing the extent of deeper injuries as well as anal sphincter rupture. However, the World Health Organization (WHO) coding system, the International Classification of Diseases (ICD), has not yet been revised accordingly and does not reflect current knowledge. This was also noted by Macedo et al., who recently also established good interrater reliability of a similar classification for second-degree tears [18]. In contrast to our findings, Macedo’s group have found no significant difference in patient reported symptoms between their classification groups [19]. The reason for this may be that their classification is limited to describing the superficial perineal body, and does not take into account the deeper structures of the pelvic floor. Also, their data concerns a smaller population, 803 women compared with our dataset of 11,203 women. The more extensive tears are rare, and a larger study population may be more appropriate regarding study power.

Most second-degree tears studied (73%) were small, 070.1X, A and B. Only 2% of the tears were reported to have an anovaginal distance (AVD) < 1 centimeter (070.1B). This corroborates the findings that an AVD of less than one centimeter is associated with anal sphincter injury and should be uncommon in second-degree tears [13]. The outcome for the rare O70.1B cases was similar to, or even better than that for O70.1 A cases, indicating correct classification and suturing.

Perineal tears reportedly heal well once they are found and sutured correctly. Our data show that women with larger second degree- tears have worse outcomes than those with smaller tears despite being registered in labor wards with a special interest in the follow-up. For follow-up, larger tears (070.1 C and D) seem more important to monitor because of the more common adverse outcomes. Concomitant levator ani injuries may be the cause of the higher rate of complications among the larger second-degree tears that required longer suturing times. The separate codes for levator ani injury are still not incorporated in the Swedish obstetric medical record systems and therefore also not yet followed in the PLR. Identification of levator ani avulsions in the delivery room is not routine and is considered difficult [20]. However, pararectal fatty tissue seen in a tear is an anatomically unequivocal sign of levator ani discontinuity, as is seeing muscle tissue visibly torn from the arcus tendineus levator ani or pubic bone. The formation of paravaginal hematoma due to bleeding from the muscle vessels is a strong predictor for avulsion [21]. It is now possible to start classifying this important aspect of obstetric injury, should it be found. Early repair of levator ani avulsion may not be possible, but follow-up and rehabilitation may benefit these women. We hope that the presence of a code will inspire early recognition, follow up and research regarding levator ani avulsions in the future.

A strength of the study is that it is the first large register study that describes patient-reported outcomes according to the extent of second-degree tears. It also reflects current clinical practice among Swedish midwives and doctors. Limitations of the study include the retrospective account of our endeavor to create new subclassifications, the need for which originated from a clinical perspective. Our efforts were put into creating and anchoring the concept. Designing a state-of-the-art consensus study may have improved the generalizability for other countries. Another limitation is that the interrater reliability was not established in a separate study. There is currently no standard for ultrasound evaluation of second-degree tears in the delivery room although measurement of the anovaginal distance with ultrasound has been tested in one study [13]. The implementation of our classification has been deemed feasible in other studies [11, 22]. The increasing use of the PLR for second degree tears at several Swedish delivery units permits systematic monitoring and future analysis of how the classification is interpreted in clinical use. This is an area for further research.

Other areas for future research include multivariate analyses of subclassified register data of second-degree tears in relation to prevention, treatment and patient-reported outcomes. This could provide valuable insights into the long-term effects of perineal tears.

## Conclusion

The implementation of detailed subclassifications for second-degree tears of the pelvic floor at delivery is a significant advancement. It underscores the importance of collaboration between obstetricians, gynecologists, midwives, and regulatory agencies in making the implementation of new classification systems possible. The subclassification for second-degree tears shows worse outcomes after larger tears and appears to be clinically relevant for both individual follow-up and future research. This not only enhances our understanding but also potentially improves care and treatment strategies for affected individuals.

## Electronic supplementary material

Below is the link to the electronic supplementary material.

Supplementary Material 1

## Data Availability

Data is available on request from the author. Databases from the Perineal Laceration register are publicly available from https://www.gynop.se/home/ after ethical approval and data retrieval permits from the research committe.

## Abbreviations

CI: Confidence interval
ICD: International Classification of Disease
ICD-10-SWE: The Swedish Edition of the ICD-10
OR: Odds ratio
PEG: Pelvic Floor Education Group
PLR: Perineal Laceration Register
SFOG: Swedish Society of Obstetrics and Gynecology

## References

[1] Rotstein E, Åhlund S, Lindgren H, Lindén Hirschberg A, Rådestad I, Tegerstedt G. Posterior compartment symptoms in primiparous women 1 year after non-assisted vaginal deliveries: a Swedish cohort study. Int Urogynecol J. 2021;32(7):1825–32. 10.1007/s00192-021-04700-6. 33646348 10.1007/s00192-021-04700-6 PMC8295137

[2] Åhlund S, Rådestad I, Zwedberg S, Lindgren H. Perineal pain the first year after childbirth and uptake of post-partum check-up- A Swedish cohort study. Midwifery. 2019;78:85–90. 10.1016/j.midw.2019.08.004. 31400597 10.1016/j.midw.2019.08.004

[3] Oversand SH, Staff AC, Sandvik L, Volløyhaug I, Svenningsen R. Levator Ani defects and the severity of symptoms in women with anterior compartment pelvic organ prolapse. Int Urogynecol J. 2018;29(1):63–9. 10.1007/s00192-017-3390-8. 28620795 10.1007/s00192-017-3390-8

[4] Andrews V, Sultan AH, Thakar R, Jones PW. Occult anal sphincter injuries–myth or reality? BJOG. 2006;113(2):195–200. 10.1111/j.1471-0528.2006.00799.x. 16411998 10.1111/j.1471-0528.2006.00799.x

[5] Faltin DL, Boulvain M, Floris LA, Irion O. Diagnosis of anal sphincter tears to prevent fecal incontinence: a randomized controlled trial. Obstet Gynecol. 2005;106(1):6–13. 10.1097/01.AOG.0000165273.68486.95. 15994610 10.1097/01.AOG.0000165273.68486.95

[6] Singh H, Connor DM, Dhaliwal G. Five strategies for clinicians to advance diagnostic excellence. BMJ. 2022;376:e068044. 10.1136/bmj-2021-068044. 35172968 10.1136/bmj-2021-068044

[7] Ogrinc G, Davies L, Goodman D, Batalden P, Davidoff F, Stevens D. SQUIRE 2.0 (Standards for quality improvement reporting Excellence): revised publication guidelines from a detailed consensus process. BMJ Qual Saf. 2016;25(12):986–92. 10.1136/bmjqs-2015-004411. 26369893 10.1136/bmjqs-2015-004411 PMC5256233

[8] Zimmo K, Laine K, Vikanes Å, Fosse E, Zimmo M, Ali H, et al. Diagnosis and repair of perineal injuries: knowledge before and after expert training-a multicentre observational study among Palestinian physicians and midwives. BMJ Open. 2017;7(4):e014183. 10.1136/bmjopen-2016-014183. 28389490 10.1136/bmjopen-2016-014183 PMC5558821

[9] Uustal E, Ekeus C, Vitols S, Lillieqvist J. Anal Sphincter injuries. A systematic review of medical, social and ethical aspects. (2016). Accessed February 26 2023. https://www.sbu.se/en/publications/sbu-assesses/anal-sphincter-injuries/

[10] Bäckenbottenutbildning.se. http://backenbottenutbildning.se/index.php/metodik/metodik (2017). Accessed August 11 2023.

[11] Edqvist M, Hildingsson I, Mollberg M, Lundgren I, Lindgren H. Midwives’ management during the second stage of labor in relation to second-Degree Tears-An experimental study. Birth. 2017;44(1):86–94. 10.1111/birt.12267. 27859542 10.1111/birt.12267 PMC5324579

[12] Hjertberg L, Uustal E, Pihl S, Blomberg M. Maternal body mass index and anovaginal distance in active phase of term labor. Biomed Res Int. 2018;2018:1532949. 10.1155/2018/1532949. 29707565 10.1155/2018/1532949 PMC5863348

[13] Pihl S, Uustal E, Blomberg M. Anovaginal distance and obstetric anal sphincter injury: a prospective observational study. Int Urogynecol J. 2019;30(6):939–44. 10.1007/s00192-018-3838-5. 30535980 10.1007/s00192-018-3838-5 PMC6511353

[14] Pihl S, Blomberg M, Uustal E. Internal anal sphincter injury in the immediate postpartum period; prevalence, risk factors and diagnostic methods in the Swedish perineal laceration registry. Eur J Obstet Gynecol Reprod Biol. 2020;245:1–6. 10.1016/j.ejogrb.2019.11.030. 31825790 10.1016/j.ejogrb.2019.11.030

[15] Otterheim M, Hjertberg L, Pihl S, Uustal E, Blomberg M. Complications 8 weeks after an obstetric second-degree perineal laceration in relation to body mass index. Int Urogynecol J. 2023. 10.1007/s00192-023-05609-y. 37584704 10.1007/s00192-023-05609-y PMC10810915

[16] Lindberg IPM, Nilsson M, Uustal E, Lindqvist M. Taken by surprise - Women’s experiences of the first eight weeks after a second degree perineal tear at childbirth. Midwifery. 2020. 10.1016/j.midw.2020.1027. 32454376 10.1016/j.midw.2020.102748

[17] Pihl K. Diagnoshandbok för Kvinnosjukvården. Sixth edition ed. Stockholm: SFOG; 2020.

[18] Macedo MD, Ellström Engh M, Siafarikas F. Detailed classification of second-degree perineal tears in the delivery ward: an inter-rater agreement study. Acta Obstet Gynecol Scand. 2022;101(8):880–8. 10.1111/aogs.14369. 35546433 10.1111/aogs.14369 PMC9564677

[19] Macedo MD, Risløkken J, Rotstein E, Benth J, Ellström Engh M, Siafarikas F. Pelvic floor symptoms according to the severity of second-degree perineal tears within 12 months post-partum: A longitudinal prospective cohort study. Acta Obstet Gynecol Scand. 2024;103(7):1366–76. 10.1111/aogs.14854. 38709004 10.1111/aogs.14854 PMC11168255

[20] Doxford-Hook EA, Slemeck E, Downey CL, Marsh FA. Management of levator Ani avulsion: a systematic review and narrative synthesis. Arch Gynecol Obstet. 2023;308(5):1399–408. 10.1007/s00404-023-06955-4. 36808288 10.1007/s00404-023-06955-4

[21] van Delft K, Thakar R, Shobeiri SA, Sultan AH. Levator hematoma at the attachment zone as an early marker for levator Ani muscle avulsion. Ultrasound Obstet Gynecol. 2014;43(2):210–7. 10.1002/uog.12571. 23893754 10.1002/uog.12571

[22] Edqvist M, Ajne G, Teleman P, Tegerstedt G, Rubertsson C. Postpartum perineal pain and its association with sub-classified second-degree tears and perineal trauma-A follow-up of a randomized controlled trial. Acta Obstet Gynecol Scand. 2024;103(11):2314–23. 10.1111/aogs.14938. 39150169 10.1111/aogs.14938 PMC11502413

