# Supplementary material for: Subclassification of second-degree tears at delivery: creation and reported outcomes
Source: BMC Pregnancy Childbirth. 2025 Mar 11;25:272. doi: 10.1186/s12884-025-07371-z (PMC11899676; doi:10.1186/s12884-025-07371-z)
Supplement: Supplementary file 1 — Supplementary Material 1 [file 12884_2025_7371_MOESM1_ESM.docx]

Supporting information subclassification article

Link to questionnaires PLR

[GynOp questionnaires - Nationella kvalitetsregistret inom gynekologisk kirurgi](https://www.gynop.se/home/gynops-questionnaire/)
